# Age-related synaptic loss of the medial olivocochlear efferent innervation

**DOI:** 10.1186/1750-1326-5-53

**Published:** 2010-11-26

**Authors:** Benjamin Fu, Colleen Le Prell, Dwayne Simmons, Debin Lei, Angela Schrader, Amelia B Chen, Jianxin Bao

**Affiliations:** 1 Department of Otolaryngology, Washington University, St. Louis, MO, 63110, USA; 2 Department of Communicative Disorders, Speech and Hearing Center, University of Florida, Gainesville, FL, 32610, USA; 3 Department of Physiological Science and the Brain Research Institute UCLA, Los Angeles, CA, 90095, USA; 4 Center for Aging, Washington University, St. Louis, MO, 63110, USA; 5 The Division of Biology & Biomedical Science and Neuroscience Program, Washington University, St. Louis, MO, 63110, USA

## Abstract

Age-related functional decline of the nervous system is consistently observed, though cellular and molecular events responsible for this decline remain largely unknown. One of the most prevalent age-related functional declines is age-related hearing loss (presbycusis), a major cause of which is the loss of outer hair cells (OHCs) and spiral ganglion neurons. Previous studies have also identified an age-related functional decline in the medial olivocochlear (MOC) efferent system prior to age-related loss of OHCs. The present study evaluated the hypothesis that this functional decline of the MOC efferent system is due to age-related synaptic loss of the efferent innervation of the OHCs. To this end, we used a recently-identified transgenic mouse line in which the expression of yellow fluorescent protein (YFP), under the control of neuron-specific elements from the thy1 gene, permits the visualization of the synaptic connections between MOC efferent fibers and OHCs. In this model, there was a dramatic synaptic loss between the MOC efferent fibers and the OHCs in older mice. However, age-related loss of efferent synapses was independent of OHC status. These data demonstrate for the first time that age-related loss of efferent synapses may contribute to the functional decline of the MOC efferent system and that this synaptic loss is not necessary for age-related loss of OHCs.

## Background

Functional decline of the nervous system is a cardinal feature of normal aging [for recent review, [1,2]]. Age-related hearing loss (presbycusis) is the third most prevalent condition of elderly persons, exceeded only by arthritis and hypertension, with approximately 97% of people experiencing a decline in hearing during aging [3,4]. Presbycusis is also characterized by reduced speech understanding in noisy environments, slowed central processing of acoustic information, and impaired sound localization. In human presbycusis, a pattern of progressive hearing loss typically starts at the high frequencies. This pattern is also observed in C57BL/6J inbred mice, a well-studied animal model for presbycusis [5]. The age-related functional decline of hearing corresponds to a loss of outer hair cells (OHCs) and spiral ganglion neurons in the basal region of the cochlea. However, little is known about possible cellular and molecular mechanisms underlying these age-related cell losses [for review, [5-8]]. Recently, age-related synaptic loss between inner hair cells (IHCs) and spiral ganglion neurons (SGNs) has been found prior to age-related SGN loss, although there is no direct link between the synaptic loss and the loss of SGN during aging [9]. Interestingly, a series of studies have clearly demonstrated an age-related functional decline of the medial olivocochlear efferent (MOC) system prior to OHC degeneration both in humans and mice [10-13]. This age-related change of the MOC efferent system could contribute to the greater difficulty experienced by elderly individuals when listening in noisy environments because the MOC most likely is evolved in "unmasking" biologically important acoustic signals by reducing the response of the cochlea to simultaneous low-level noises [14-16]. However, the possible cellular and molecular mechanisms underlying this functional decline are unknown.

The auditory efferent system to the cochlea consists of two major divisions: the MOC pathway and the lateral olivocochlear (LOC) pathway [17-19]. The LOC system originates in the lateral nuclei of the superior olivary complex and forms axodendritic synapses on afferent dendrites of type-I spiral ganglion neurons below IHCs. Although function of the LOC system is not well understood, it may contribute to the modulation of auditory nerve activity [20,21]. The MOC pathway, comprising thick myelinated nerve fibers, originates from neurons within medial and ventral regions of the superior olivary complex and makes large axosomatic synapes with OHCs (DAdditional file 1: Diagram 1). This innervation can be demonstrated by cholinergic markers [22,23]. Extensive studies have clearly demonstrated that the activation of the MOC system attenuates cochlear responses to acoustic stimulation [24-33]. This attenuation is convincingly shown arising from cholinergic innervations of the MOC synapses on OHCs via the α9 nicotinic cholinergic receptors [15,34]. In both humans and animal models, the strength of the MOC efferent innervation can be measured by the degree of contralateral attenuation of otoacoustic emissions (OAE), particularly distortion product OAEs (DPOAES) [35-38], or ipsilateral DPOAE adaptation [39,40]. Using in vivo functional assays, age-related functional decline of the MOC system has been well documented both in human and animals [10-13,41]. However, it is unclear whether this age-related functional decline is due to age-related loss of OHC or loss of MOC synapses on OHCs. Recently, we has discovered that MOC terminals on OHCs can be visualized in one line of transgenic mice in which the expression of yellow fluorescent protein (YFP) is under the control of neuron-specific elements from the thy1 gene [42]. Therefore, this animal model provided us with an opportunity to directly address whether there is an age-related loss of MOC terminals in the cochlea, and whether this loss occurs prior to the loss of OHCs.

## Results

### Characterization of YFP-12 mice for the MOC synapses on OHCs

**Figure 1:**
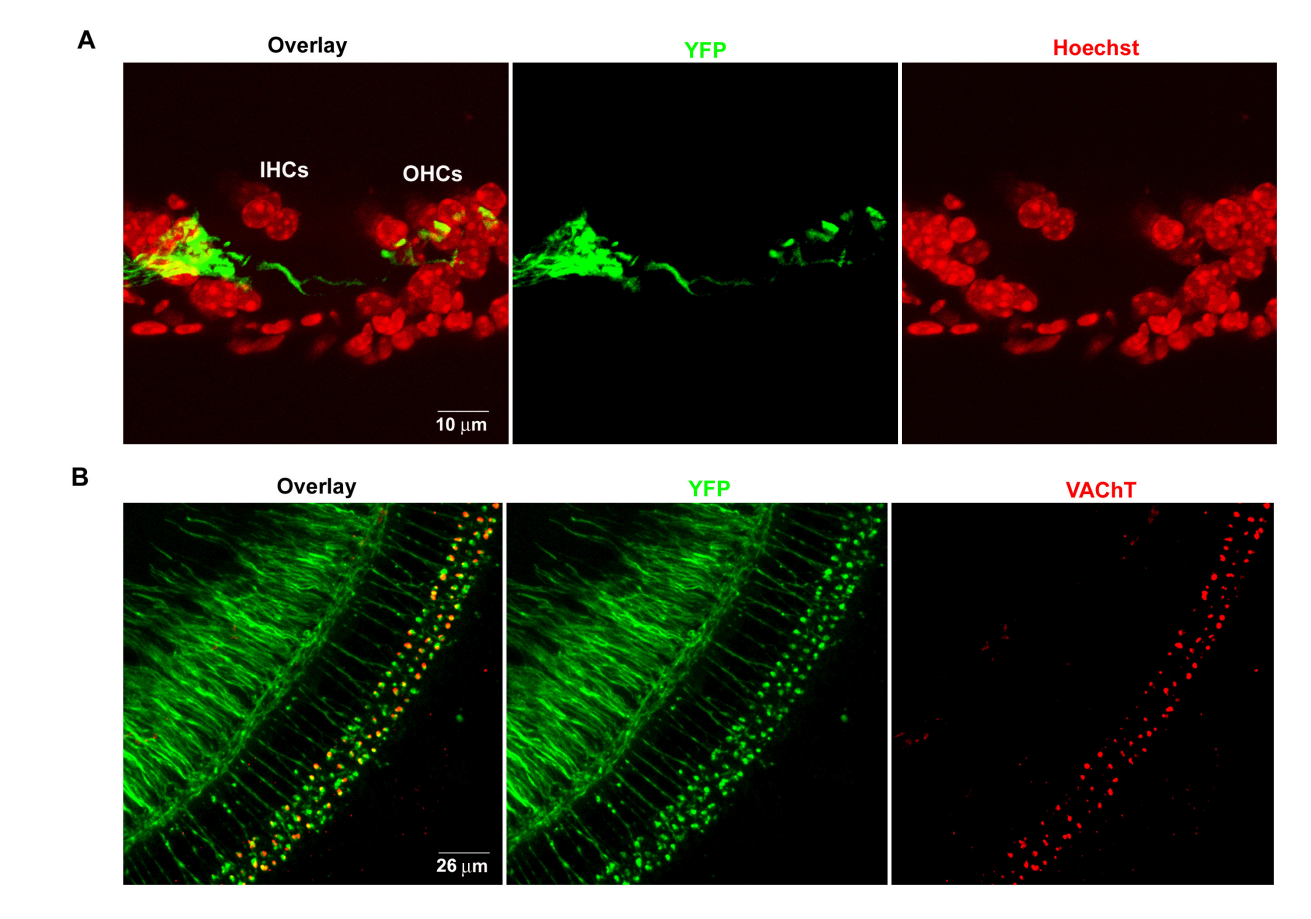
**The MOC efferent innervation in the cochlea of YFP-12 transgenic mice**. (*A) *A sagittal cochlear section from one 2-month old YFP-12 mouse with the Hoechst staining for nuclei (red) and YFP (green). (*B) *A wholemount cochlea from one 2-month-old YFP-12 mouse immunostained with an antibody against VAChT (Red).

Certain neuronal populations and their synaptic terminals are well labeled in transgenic mouse lines with Yellow Fluorescent Protein (YFP) expression under the control of neuron-specific elements from the thy1 gene [42] We examined four such transgenic lines and discovered one line, the YFP-12 line, which has well-labeled synaptic terminals underneath both the IHCs and the OHCs (Figure 1A). OHCs receive synaptic innervation from both MOC efferent neurons and type-II SGNs. These YFP-positive terminals were thus immunostained with an antibody against VAChT, a reliable cholinergic marker for MOC synapses, to determine the extent of these synapses. From the whole-mount horizontal sections, we observed a substantial overlap between the distribution of YFP-positive and VAChT-positive synapses on OHCs (Figure 1B).

**Figure 2:**
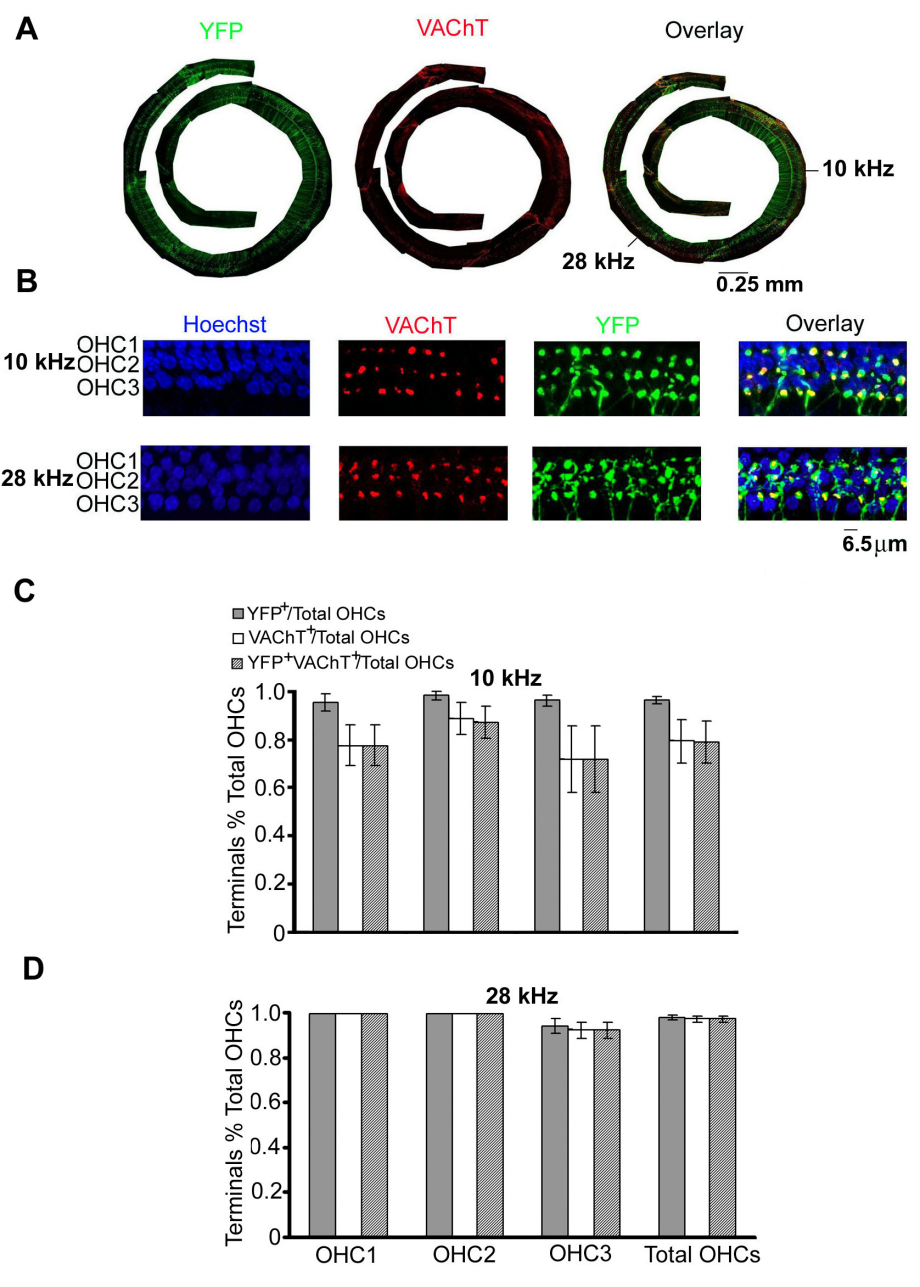
**Quantification of YFP- and VAChT-positive synapses at the OHC regions at 2 months old**. (A) The whole cochlea from base to apex. The exact locations for 10 and 28 kHz are indicated on the overlay panel after mapping. (B) High power micrographs at 10 (upper panel) and 28 kHz (low panel) of OHC areas. The rows of OHCs are numbered OHC1, OHC2, and OHC3. (C) Quantification data for each row of OHCs at 10 kHz (data from 5 animals). (D) Quantification data for each row of OHCs at 28 kHz (data from three animals). Mean values (+/- standard error of the mean) were obtained by averaging counting data from an average of 15 OHCs per row.

To precisely determine whether each YFP-positive terminal was also VAChT-positive (whole length of the cochlear spiral shown in Figure 2A), we focused on two frequency regions during which presbycusis proceeds at different rates [5,43]. Based on previous findings of DPOAE and contralateral DPOAE suppression tests [44], age-related loss of MOC function in C57BL/6J mice begins around 2 months of age. Since this age-related functional decline is much smaller at 10 kHz than at 20 to 30 kHz, we focused on the 10 kHz and 28 kHz regions of the cochlea. Confocal images at these two frequency regions are shown in Figure 2B. Nearly all YFP-positive terminals were also VAChT-positive at both 10 and 28 kHz regions in the 2-month-old mice; however, a few YFP-positive terminals below OHCs did not colocalize with VAChT. We counted both YFP-positive and VAChT-positive terminals below OHCs at the 10 kHz and 28 kHz regions (about 45 OHCs for each region). At the 10 kHz region (Figure 2C), the ratio of YFP-positive terminals to OHCs was about 0.99 for the first row of OHCs (OHC1) and 0.98 for the second and third OHC rows. The ratio of VAChT-positive terminals to OHCs was about 0.87 for the first OHC row, 0.96 for the second row, and 0.85 for the third row. The ratio of VAChT-labeled terminals and YFP-VAChT double labeled terminals were the same, suggesting that all VAChT labeled terminals colocalized with YFP. Only about one or two YFP-positive terminals per 45 OHCs did not appear together with VAChT. This could be the result of innervations from type-II SGNs or MOC fibers not expressing VAChT. At the 28 kHz region (Figure 2D), virtually all YFP-labeled terminals below OHCs co-localized with VAChT and all OHCs had at least one YFP-labeled terminal. The ratio between YFP- and VAChT-positive terminals and OHCs was 1.00 for the first and second rows of OHCs. The ratio was also 1.00 for two out of three mice for the third row of OHCs. In one case, there were no YFP- or VAChT-positive terminals below two OHCs.

### Age-related loss of the MOC synapses in the cochlea

**Figure 3:**
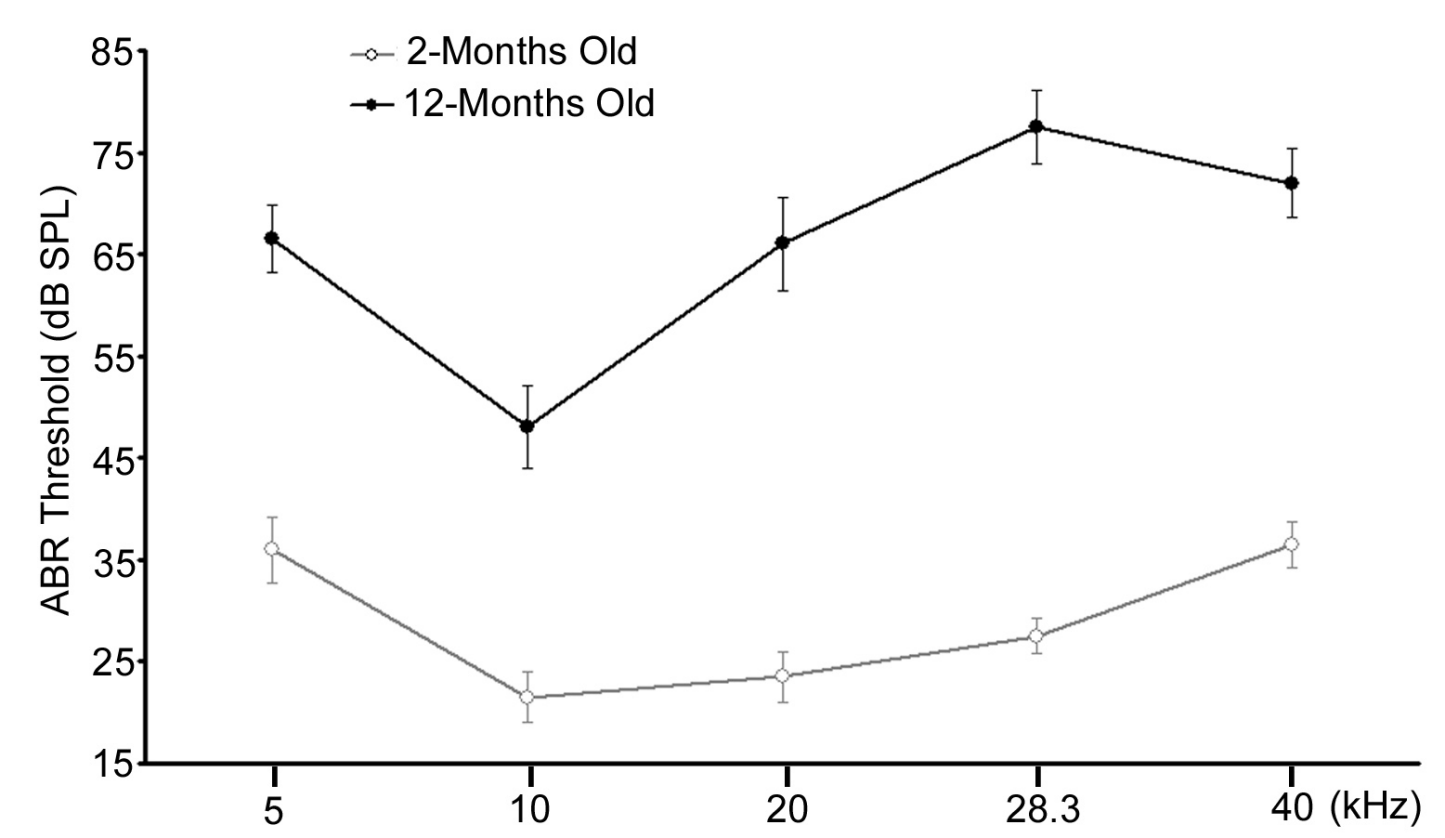
**ABR thresholds for 2- and 12-month-old YFP-12 mice under the C57BL/6J genetic background**. ABR thresholds (Mean ± S.D) for 2-month-old mice (n = 7; grey line) were significantly lower than the thresholds (Mean ± S.D) from 12-month-old mice (n = 7; black line).

Prior to examining possible age-related loss of MOC efferent synapses underneath OHCs in animals from the YFP-12 transgenic line, audio brainstem responses (ABRs) were conducted on young (2-month-old) and old (12-month-old) YFP-12 mice (Figure 3). Animals at both young and old ages had the lowest hearing thresholds at 10 kHz, with increasing thresholds for higher frequencies. Although all frequency regions experienced elevated thresholds due to age, as expected for C57BL/6J mice, greater hearing loss occurred at higher frequencies than at lower frequencies. The ABR threshold shift between 2-month-old and 12-month-old mice at 10 kHz region was approximately 26 dB (*p *= 2.79 × 10^-5^), while the shift was about 50 dB at 28 kHz *(p = *6.73 × 10^-9^).

**Figure 4:**
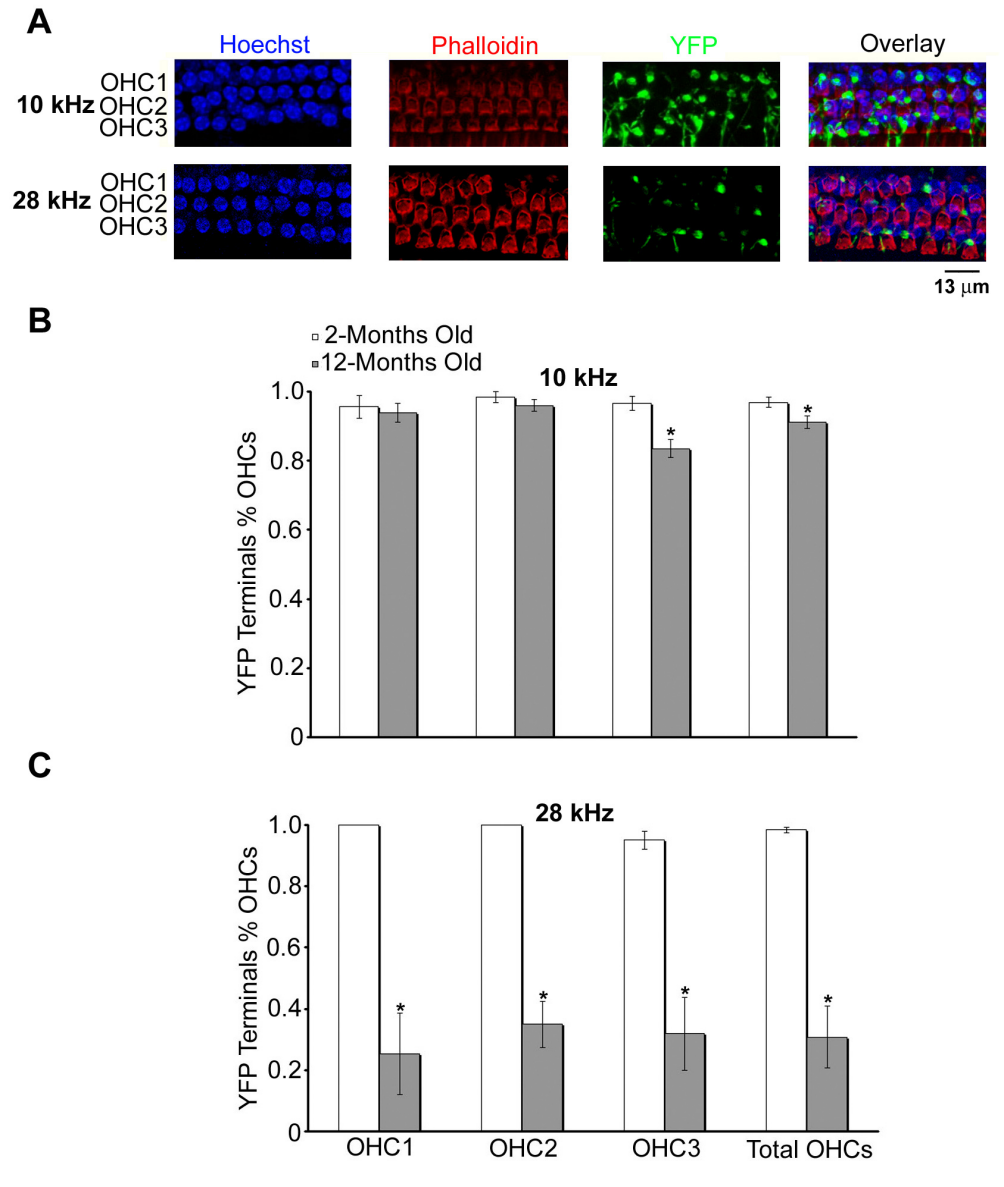
**Age-related loss of the MOC synapses**. (A) OHCs were labeled with both Hoechst and phalloidin, and the MOC synapses with YFP. (B) Quantification data for 2- and 12-month-old mice at 10 kHz (data from three animals for each age group). (C) Quantification data for 2- and 12-month-old mice at 28 kHz (data from three animals for each age group). Mean values (+/- standard error of the mean) were obtained by averaging counting data from an average of 15 OHCs per row.

In order to assess age-related loss of the MOC synapses below OHCs, confocal images were taken of the 10 and 28 kHz frequency regions in 12-month-old mice (Figure 4A). To monitor possible loss of OHCs, we also labeled OHC bundles with phalloidin. At the 10 kHz region, there was no obvious OHC loss, and the distribution of YFP-positive synapses underneath OHCs was comparable to mice at 2 months. At the 28 kHz region, age-related loss of OHCs was minimal. However, a substantial decrease of YFP-positive terminals was observed in this region, in spite of survival of OHCs. We quantified the number of YFP-positive terminals for 2- and 12-month-old mice at the 10 and 28 kHz regions. In the 10 kHz region (Figure 4B), the only significant (p < 0.005) difference between 2- and 12-month-old mice in the number of YFP-positive synapses was found in the third row of OHCs. There was roughly a 13% decline in efferent terminals at the third row of OHCs. In contrast to the 10 kHz region, the 28 kHz region demonstrated a more dramatic decline in efferent synapses among all OHC rows in 12-month-old mice (Figure 4C). Age-related loss of the MOC synapses was 75% in the first row, 65% in the second row, and 63% in the third row.

### No correlation between age-related OHC loss and the loss of MOC synapses

**Figure 5:**
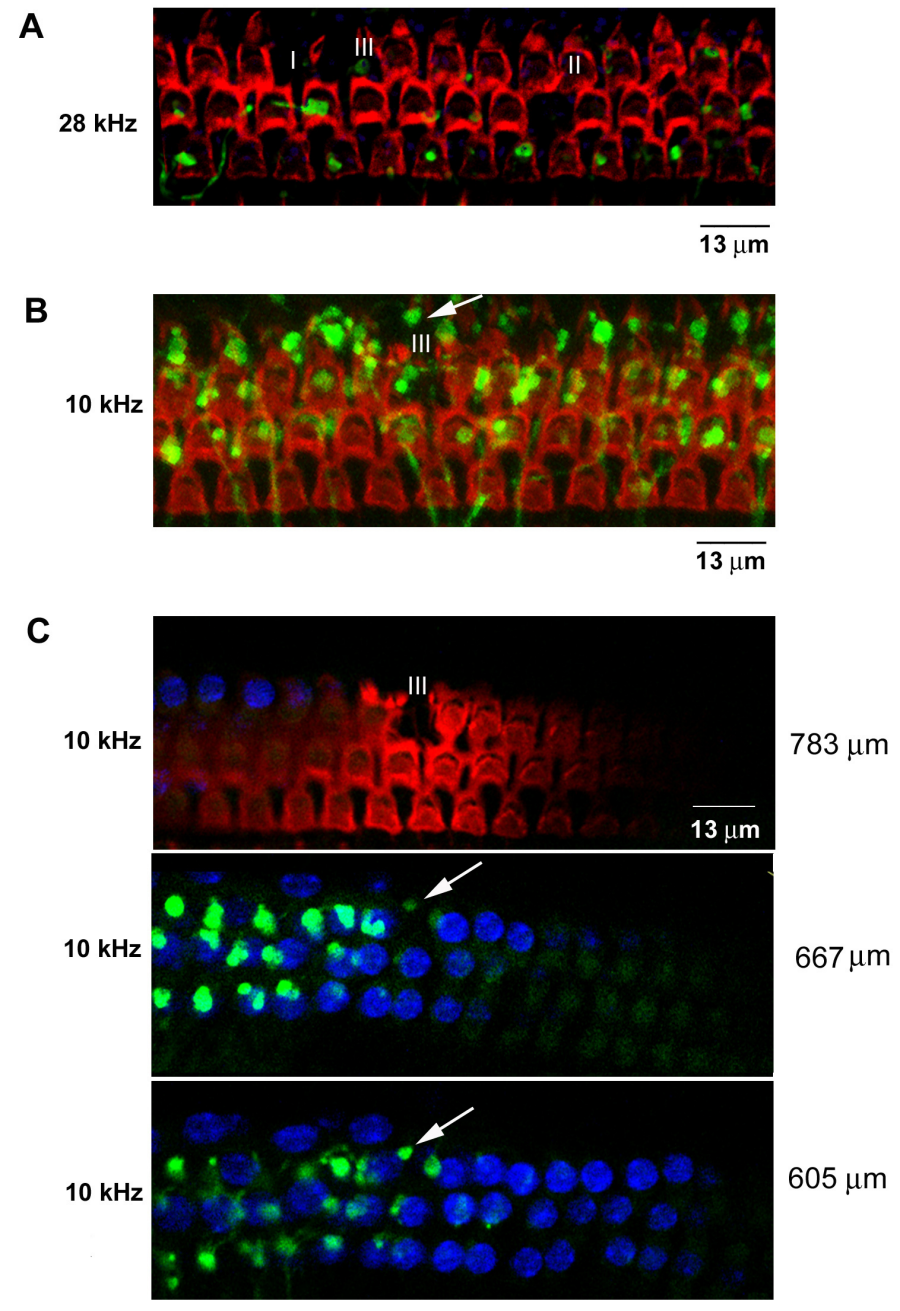
**Correlation between age-related loss of OHC and the MOC synapses**. (A) One overlay of confocal images at 28 kHz from one 12-month-old YFP-12 mouse with the phalloidin staining for hair bundles (red) and YFP (green). Missing MOC synapse and OHCs were easily detected. (B) One overlay of confocal images at 10 kHz from one 12-month-old YFP-12 mouse with the phalloidin staining for hair bundles (red) and YFP (green). One missing OHC was observed. (C) These panels are from the same regions as (B). The top panel shows the layer of hair bundles and the appearance of OHC nuclei on the left side, which is closer to the lens than the right side. The middle and bottom panels show the MOC synapse at the location of one missing OHC.

By using a confocal microscope to scan the whole-mount cochlear sections from the top of hair bundles to the nuclei of Deiters' cells at an interval of 0.96 μm, we observed three types of age-related loss of MOC synapses (Figure 5A). The first type was a loss of both OHCs and the MOC synapses that would have previously contacted the missing OHCs. The second was a loss of MOC synapses despite the presence of intact OHCs, which suggests that either survival of OHCs is not dependent on MOC innervation or MOC synaptic degeneration was recent enough that OHC survival had not yet been compromised. The third type of age-related change was the presence of MOC synapses despite OHC death. Because of the 3-D nature of the organ of Corti, it was initially difficult to distinguish whether the MOC synapse was at the previous level of the OHC or if it had withdrawn to the level of the Deiters' cells. Thus, we examined the third type in more detail. In Figure 5B, an overlay at the 10 kHz region clearly shows one missing OHC with an MOC synapse - the result of the angle of OHCs at the 3-D overlay. We then focused on the compact OHC nuclei as reliable markers, since they lie closer to MOC synapses. After examining every optical section at about 116 μm from the hair bundles, the MOC synapse appeared underneath the missing OHC, and its intensity was similar to the MOC innervation on the right neighbor OHC (The middle panel of Figure 5C). Thus, the loss of OHCs and loss of MOC synapses could each occur independently.

## Discussion

Possible causes of age-related functional decline of both the central and peripheral nervous system (CNS and PNS) are still not completely understood. Because extensive neuronal death is observed in age-related neurodegenerative diseases, it was proposed that neuronal death might also contribute to normal age-related functional decline of the nervous system [45-47]. However, in the CNS, loss of synapses rather than loss of neurons may be the major cause of age-related functional decline [48-50]. In the PNS, however, age-related loss of both synapses and neurons significantly contributes to functional decline [51-53]. In the cochlea, age-related loss of hair cells and spiral ganglion neurons are a major contributor to presbycusis although possible cellular and molecular mechanisms underlying the death of these cells are still unknown [2,4,45]. Recent data have strongly suggested the contribution of the MOC efferent system to age-related hearing loss [10-13,41]. In the present study, our results have showed for the first time that age-related loss of efferent terminals on OHCs occurs in C57BL/6J mice. Furthermore, we have found that age-related loss of the efferent OHC terminals and OHCs can occur independently, which suggests possibly a separate mechanism contributing to the age-related changes of these two biological structures.

In the cochlea, previous studies have clearly demonstrated synaptic loss between SGNs and IHCs, and withdrawal of SGN afferent fibers occur prior to SGN death during aging [9,54,55]. These data imply that SGN degeneration starts at its synapse with IHCs, and progress toward the cell body [9], although no evidence is currently able to prove conclusively that this synaptic loss was the actual cause of age-related SGN loss. In the MOC efferent system, extensive data collected from non-invasive functional testing clearly demonstrated age-related functional decline of the MOC system. Particularly, contralateral suppression of DPOAEs by the MOC efferent system is absent at middle (15 to 30 kHz) and high (30 - 45) frequencies in C57BL mice by eight weeks old [13]. Interestingly, our finding shows that every OHC still receives the MOC innervation at the 28 kHz region at 2 months old. This suggests that MOC neurotransmission at this synapse may be nonfunctional. Because most of the terminals are lost by 12 months old, it is possible that the MOC efferent synapses may lose function such as synaptic transmission before they are eliminated with aging. If this scenario is true, we would expect nearly all of MOC efferent terminals to be present even at 22 months in CBA/CaJ mice because the MOC suppression just starts to differ from young animals [12]. Thus, in the future, it would be necessary to place the YFP-12 transgene into the CBA/CaJ genetic background.

Morphologically, it seems that age-related loss of the MOC efferent terminals does not depend on OHCs because most of the terminal loss occurs prior to age-related loss of OHCs. However, age-related functional changes in hair cells could contribute to this dramatic loss of MOC terminals in C57BL/6J mice. For example, studies have shown that abnormal tip link structure of the hair bundle results from a cadherin 23 mutation in C57BL/6J mice and may lead to a defective mechanoelectrical transduction apparatus [56]. This abnormal mechanoelectrical transduction may lead to or be associated with cumulative changes in either synaptic transmission between OHCs and efferent terminals or in abnormalities that affect slow motility but not the prestin-based electromotility. The presence of a few MOC terminals without OHCs at the 28 kHz region from 12 months old mice seems to suggest that efferent terminals can survive the loss of OHCs. If similar phenomena are also observed in CBA/CaJ mice, it would further indicate that age-related loss of OHCs and the MOC terminals are two independent processes.

## Conclusion

Extensive synaptic and neuronal loss contributes to the pathology of age-related neurodegenerative diseases such as Alzheimer's disease [50,57]. It is important to address the question of whether synaptic loss during aging is the cause or the result of neuronal loss. Our data suggest also a third possibility, a parallel independent biological process for age-related loss of neurons and synapses. Because neuronal aging is the common predisposing factor for neurodegenerative diseases, this possibility is worth exploring. In addition, our data provide the first morphological basis for age-related functional decline of the MOC efferent system in the cochlea: age-related loss of the MOC terminals.

## Methods

### Animals

The YFP-12 transgenic line was kindly provided by Joshua Sanes. This line was back-crossed over 10 generations to the C57BL/6J genetic background. Mice were housed five per cage with food and water available. They were maintained in a noise-controlled environment on a 12 hr light/dark cycle, with light onset at 6:00 a.m. All procedures here followed NIH guidelines and were approved by the animal care and use committee of Washington University.

### ABR Recording

Mice were anesthetized (80 mg/kg ketamine, 15 mg/kg xylazine, i.p.) and positioned dorsal side up in a custom headholder. Core temperature was maintained at 37°C using a thermostatically controlled heating pad in conjunction with a rectal probe (Yellow Springs Instruments Model 73A). Platinum needle electrodes (Grass) were inserted subcutaneously just behind the right ear (active), at the vertex (reference), and in the back (ground). Electrodes were led to a Grass P15 differential amplifier (0.1-10 kHz, X100), to a custom broadband amplifier (0.1-10 kHz, X1000), then digitized at 30 kHz using a Cambridge Electronic Design micro1401, in conjunction with SIGNAL and custom signal averaging software, operating on a 120 MHz Pentium PC. Sine wave stimuli generated by a Hewlett Packard 3325a digital oscillator were shaped by a custom electronic switch to 5 ms total duration, including 1 ms rise/fall times. The stimuli were amplified by a Crown D150A power amplifier and led to an Alpine SPS-OEOA coaxial speaker located 10 cm directly lateral to the right external auditory meatus. Stimuli were presented free field and calibrated using a B&K 4135 ¼ inch microphone placed where the pinna would normally be. Toneburst stimuli at each frequency and level were presented 1000 times at 20/s. The minimum sound pressure level required for a response (short-latency negative wave) was determined at 5.0, 10.0, 20.0, 28, and 40.0 kHz, using a 5 dB minimum step size. The sound level was increased in 5 dB steps and terminated at 101 dB.

### Histological Analysis

Mice were perfused transcardially with cold 2% paraformaldehyde and 2% glutaraldehyde in phosphate-buffered saline (PBS). Each cochlea was rapidly isolated, immersed in the same fixative, and the stapes immediately removed. Complete infiltration of the cochlea by fixative was ensured by making a small hole at the apex of the cochlear capsule and gently circulating the fixative over the cochlea using a transfer pipette. After overnight decalcification in sodium EDTA, cochleae were embedded in an agarose mold and cut in 200 μm slices perpendicular to the cochlear axis. Cochlear slices were mounted on a glass slide with a coverslip using Mount-quick "AQUEOUS" (Daido Sangyo Co. Ltd. Japan). The entire length of the cochlea was measured using a stereological analysis program (StereoInvestigator, MicroBrightField Inc.) on a computer connected to a Nikon Eclipse TE2000-U inverted microscope. To obtain a consistent measurement, we set the focus on the top of inner pillar cells for each section. After the whole length was obtained, a frequency-place map for the cochlea was calculated based the published formula [13]. Sections containing 10 kHz and 28 kHz regions (about 42.5% and 70% from the apex respectively) were processed for immunocytochemistry.

### Immunocytochemistry

Immunostaining was carried out similar to our previous studies [58] Cochlear sections were washed with 1× PBST (0.1 M PBS in 0.1% Tween 20) three times 10 minutes each and blocked with 10% normal goat serum (NGS; company) in 1× PBST for one hour. The sections were incubated with a rabbit antibody against the vesicular acetylcholine transporter (VAChT) (Sigma, St. Louis, MO) at 1:200 dilution in 3% NGS overnight at 4°C. The sections were then washed four times 10 minutes each in PBST. A 1:250 solution of goat anti-rabbit-CY3 was then added. Cochlear slices were washed with PBST four more times before mounting. In certain cases, the sections were incubated with phalloidin (1:200) and Hoechst (1:1000) for an hour. Again, the sections were washed with PBST four times ten minutes each prior to mounting.

### Imaging and Analysis of Data

A confocal microscope (Bio-Rad Radiance 2000 MP) was used to scan the whole cochlea section at 20× first to identify the two specific frequency regions (10 and 28 kHz), and then was switched to 40× to collect images. The images were colorized and merged using the Volocity program. The MOC synaptic terminals and hair cells were quantified for each of the three OHC rows at the 10 kHz and 28 kHz regions based on confocal images. Statistical computations were performed by Microsoft Excel.

## Conflicts of interests

The authors declare that they have no competing interests.

## Authors' contributions

BF, DL, AS and JB designed the experiments, statistical analysis, interpreted the results and drafted the manuscript. BF, DL, AS and ABC carried out the experiments. CLP, DS and JB drafted the manuscript. All authors read and approved the final manuscript.

## Supplementary Material

Additional file 1 **Diagram 1: The Medial Olivocochlear Efferent Innervation in the Cochlea**. Schematic cross section of the cochlea shows the organ of Corti. Type-I spiral ganglion neurons innervate the inner hair cell, and the outer hair cells are innervated by both type-II spiral ganglion neurons and the medial olivocochlear efferent fibers. Click here for file
