# Supplementary figures and images for: Age-related synaptic loss of the medial olivocochlear efferent innervation
Source: Mol Neurodegener. 2010 Nov 26;5:53. doi: 10.1186/1750-1326-5-53 (PMC3000387; doi:10.1186/1750-1326-5-53)

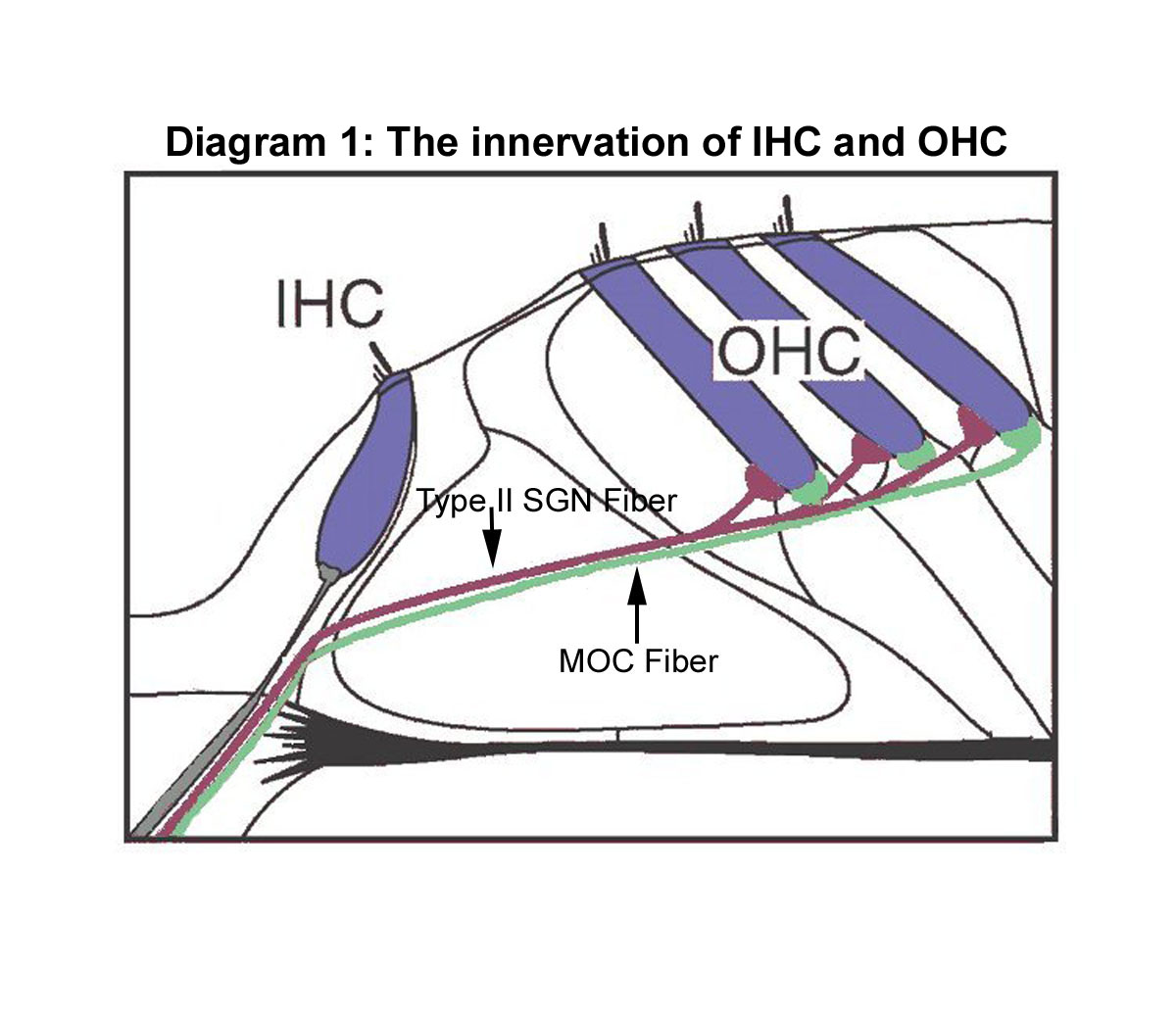

Supplement: Additional file 1 — Diagram 1: The Medial Olivocochlear Efferent Innervation in the Cochlea. Schematic cross section of the cochlea shows the organ of Corti. Type-I spiral ganglion neurons innervate the inner hair cell, and the outer hair cells are innervated by both type-II spiral ganglion neurons and the medial olivocochlear efferent fibers. [file 1750-1326-5-53-S1.JPEG]
